# Thrombus‐Induced Prosthetic Valve Dysfunction Demonstrating Hypo‐Attenuated Leaflet Thickening: A Case Report

**DOI:** 10.1002/ccr3.73518

**Published:** 2026-09-10

**Authors:** Takanori Kono, Kazuyoshi Takagi, Yusuke Shintani, Hiroki Mine, Eiki Tayama

**Affiliations:** ^1^ Division of Cardiovascular Surgery, Department of Surgery Kurume University Fukuoka Japan

**Keywords:** cardiac computed tomography, hypo‐attenuated leaflet thickening, prosthetic valve dysfunction, subclinical leaflet thrombosis, surgical aortic valve replacement

## Abstract

Hypo‐attenuated leaflet thickening (HALT) is an important and potentially underrecognized cause of late bioprosthetic valve dysfunction after surgical AVR. Multimodal imaging, including echocardiography and electrocardiogram‐gated computed tomography, is essential for diagnosis, and timely surgical or medical intervention can effectively restore valve function and improve clinical outcomes.

## 1 Introduction

Hypo‐attenuated leaflet thickening (HALT), identified as low‐attenuation regions on bioprosthetic valve leaflets detected on electrocardiogram‐gated computed tomography (CT), has been reported in approximately 4%–38% of post‐ transcatheter aortic valve implantation (TAVI) cases [1, 2]. Although HALT was initially considered a complication specific to TAVI, it is now recognized to occur after surgical aortic valve replacement (SAVR) as well [3]. HALT often remains asymptomatic and might not be accompanied by increased transvalvular gradients or elevated flow velocities. The clinical relevance of HALT remains debated. Here, we report a case of redo aortic valve replacement, in which HALT was identified on preoperative electrocardiogram‐gated CT and thrombotic valves were confirmed intraoperatively.

## 2 Case History/Examination

An 81‐year‐old man underwent aortic valve replacement (AVR) with a 25‐mm INSPIRIS RESILIA valve (Edwards Lifesciences LLC, Irvine, CA, USA) and pulmonary vein isolation for severe aortic regurgitation (AR) and paroxysmal atrial fibrillation (PAf) in April of Year X–3. His postoperative course was uneventful, and he was transferred to another hospital for rehabilitation on postoperative day 24. At discharge, echocardiography showed a peak velocity (PV) of 2.2 m/s, peak pressure gradient (PG) of 18.6 mmHg, mean PG of 11.3 mmHg, aortic valve area (AVA) of 2.5 cm^2^, and trivial mitral regurgitation (MR) and tricuspid regurgitation (TR).

**FIGURE 1:**
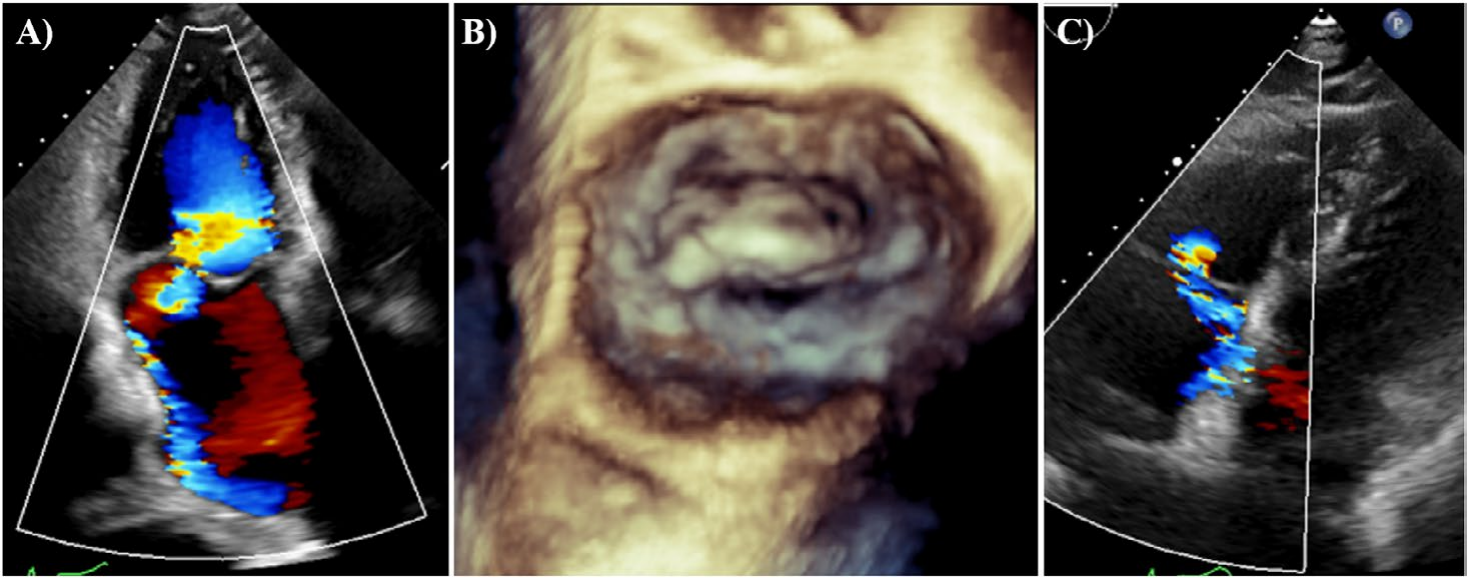
Transesophageal echocardiography (TEE) displaying severe mitral regurgitation (A). Three‐dimensional TEE demonstrating the prolapse of the A1‐2 segments (B). TEE revealing moderate tricuspid regurgitation (C).

He continued follow‐up at a local clinic. The last echocardiogram at our institution, three months after surgery, revealed a PV of 3.0 m/s and a mean PG of 18.6 mmHg. He remained asymptomatic without signs of heart failure thereafter. Around April of Year X, the patient developed lower‐leg oedema, and echocardiography demonstrated an increased aortic valve velocity (PV 3.8 m/s). Previously unrecognized severe MR and moderate TR were also identified. He was referred to our hospital in July, and a detailed evaluation was performed during hospitalization in August. Severe MR due to A1‐A2 segment of the anterior leaflet prolapse, moderate TR (Figure 1A–C), and suspected prosthetic aortic valve dysfunction were noted, and surgery was scheduled for September.

## 3 Differential Diagnosis, Investigations and Treatment

### 3.1 Differential Diagnosis

The differential diagnosis of prosthetic valve dysfunction included structural valve degeneration, prosthetic valve thrombosis, pannus formation, infective endocarditis, and patient‐prosthesis mismatch.

### 3.2 Investigations

**FIGURE 2:**
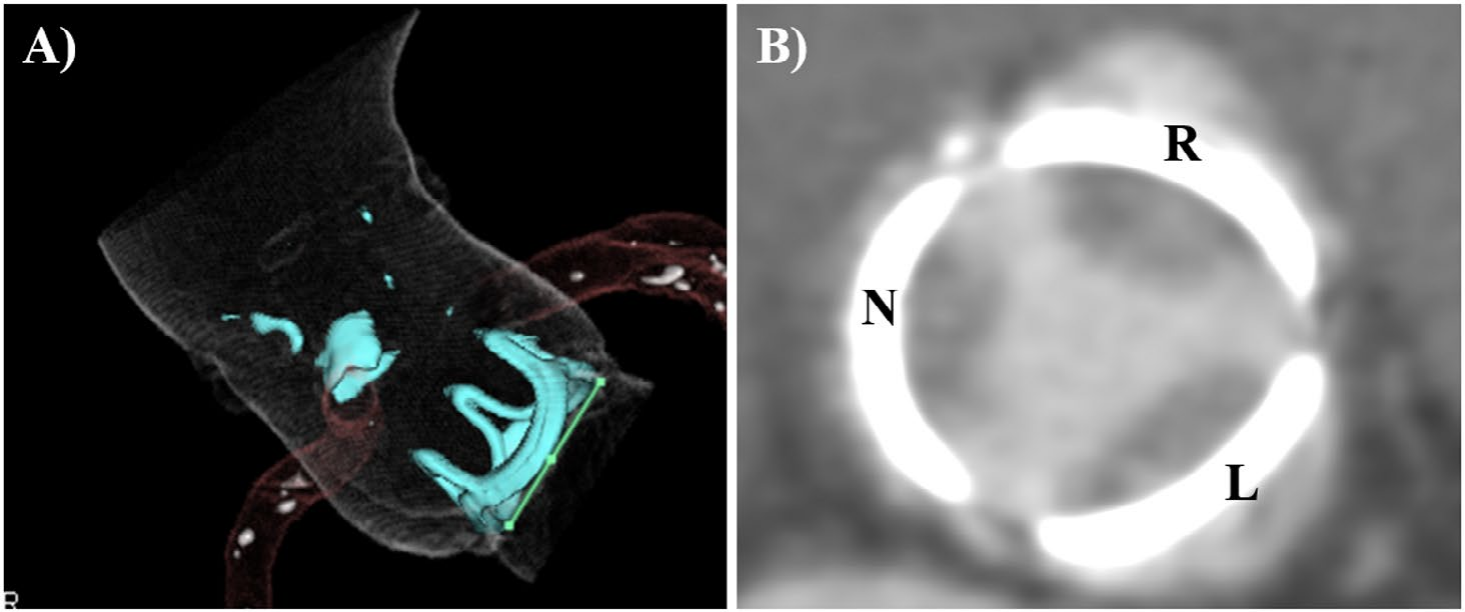
Three‐dimensional contrast‐enhanced computed tomography (CT) indicating a bioprosthetic aortic valve (A). Electrocardiogram‐gated CT demonstrating hypo‐attenuated leaflet thickening involving all three prosthetic valve leaflets (B).

**FIGURE 3:**
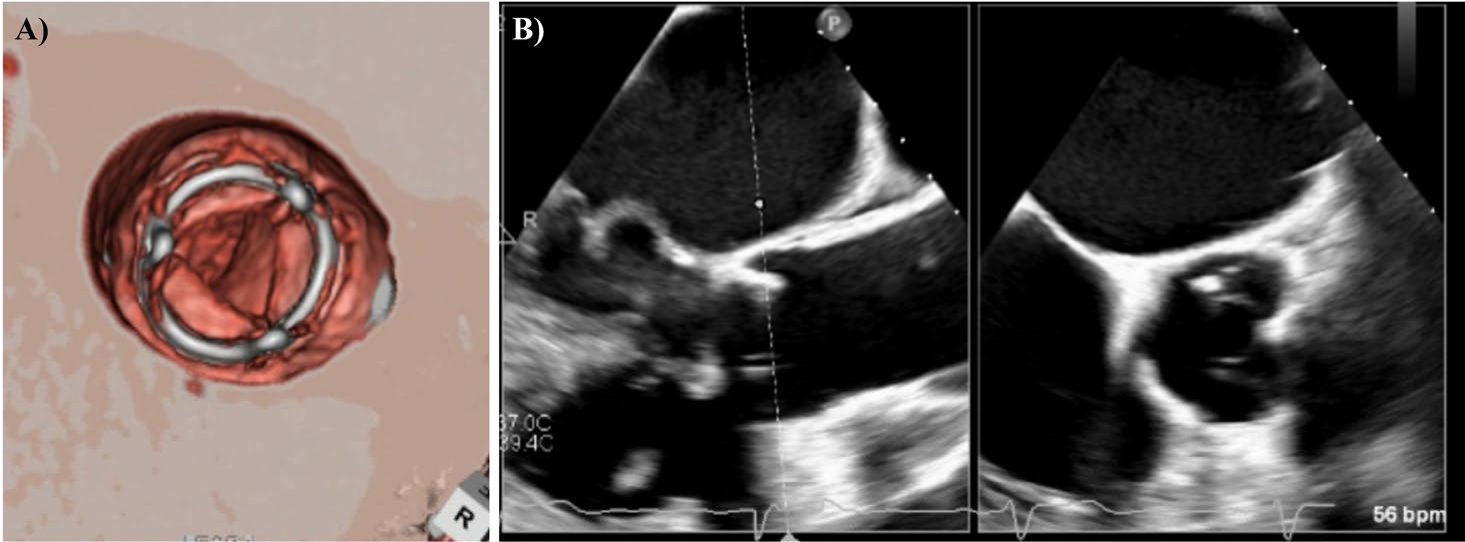
(A) Representative still image from Video S1 showing three‐dimensional reconstructed CT of the bioprosthetic aortic valve. (B) Representative transesophageal echocardiographic image from Video S2 showing the bioprosthetic aortic valve.

Preoperative electrocardiogram‐gated CT revealed HALT involving all three cusps of the bioprosthetic aortic valve (Figure 2A,B). However, although HALT was evident, three‐dimensional reconstructed CT images did not clearly demonstrate restricted leaflet motion (RLM) (Video S1 and Figure 3A). Echocardiography showed left ventricular diastolic diameter and systolic diameter (LVDd/LVDs) 54/34 mm, ejection fraction (EF) 66%, PV 3.4 m/s, peak PG 46.2 mmHg, mean PG 25.0 mmHg, and AVA 1.15 cm^2^. However, despite the elevated transvalvular gradients, transesophageal echocardiography did not provide definitive evidence of RLM of the bioprosthetic valve (Video S2 and Figure 3B). The patient had no recurrence of PAF and had been maintained on aspirin monotherapy after discontinuation of warfarin three months postoperatively.

### 3.3 Treatment

**FIGURE 4:**
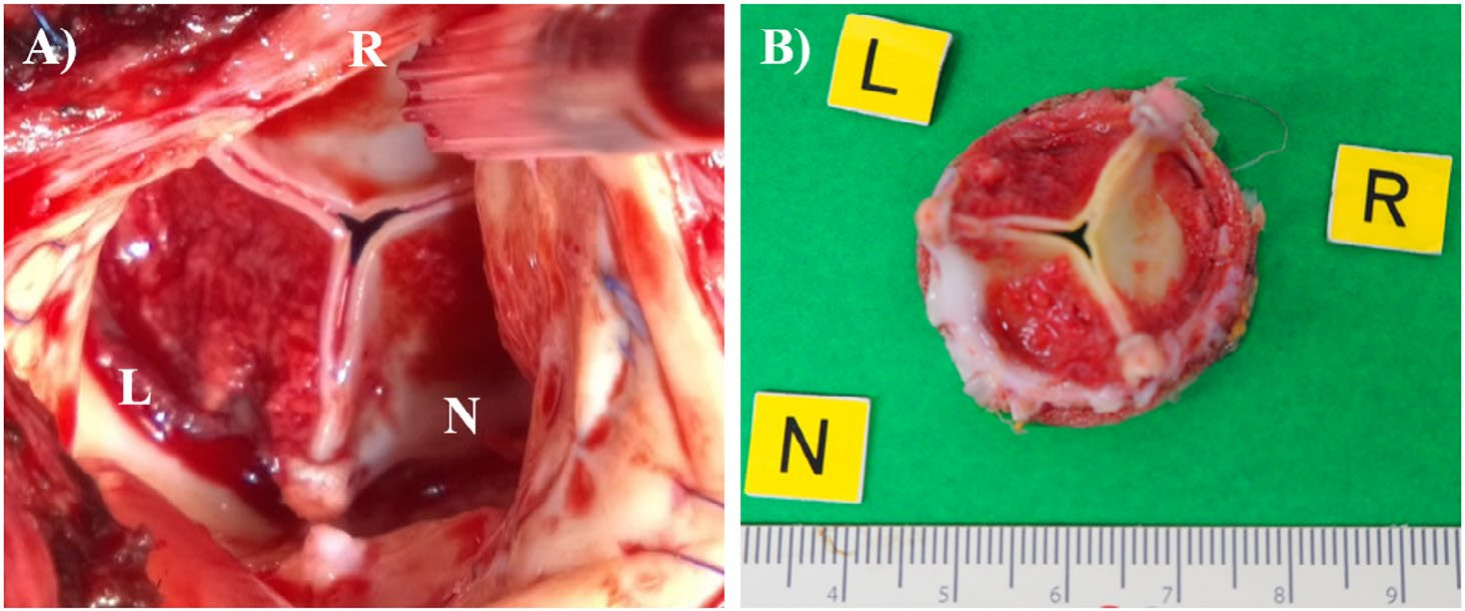
Intraoperative findings revealing thrombus formation in all three leaflets (A). Explanted prosthetic valve (B).

Intraoperatively, substantial thrombus deposition was observed on the left coronary cusp (LCC) and non‐coronary cusp (NCC), resulting in reduced leaflet mobility. Although thrombus was also present on the right coronary cusp (RCC), the extent of thrombus deposition was limited and did not appear sufficient to significantly restrict leaflet motion. Therefore, redo AVR was deemed necessary (Figure 4A,B). Redo AVR with a 25‐mm INSPIRIS RESILIA valve, mitral valve plasty using artificial chordae and a CG Future band (Medtronic Inc., Minneapolis, MN, USA), and tricuspid annuloplasty with a 30‐mm Physio Tricuspid ring (Edwards Lifesciences, Irvine, CA, USA) were performed.

### 3.4 Outcome and Follow‐Up

The patient's postoperative course was uneventful. Postoperative echocardiography demonstrated LVDd/Ds 47/31 mm, EF 65%, PV 1.9 m/s, peak PG 14.4 mmHg, mean PG 7.0 mmHg, and AVA 2.42 cm^2^. He was transferred to another hospital for rehabilitation on postoperative day 13. At the 3‐year follow‐up after redo surgery, the patient remained clinically stable without evidence of recurrent prosthetic valve dysfunction, thromboembolic events, or structural valve deterioration.

## 4 Conclusion

This report describes the coexistence of HALT and surgically confirmed thrombotic leaflet changes rather than proving causality. We believe that this case highlights the need for further studies investigating the long‐term pathological and clinical significance of HALT.

## 5 Discussion

HALT has traditionally been considered a frequent finding after TAVI; however, we and others have reported that HALT might also occur after SAVR at a frequency comparable to that observed after TAVI [3, 4]. Nevertheless, the clinical significance of HALT remains incompletely understood. Iwata et al. reported that HALT was detected in 15.2% of patients during a 5‐year follow‐up after TAVI, and found no significant differences in the incidence of structural valve degeneration (SVD) between patients with and without HALT (14.7% vs. 17.9%, respectively) [5]. In addition, no significant difference in all‐cause mortality was observed (52.9% vs. 60.0%) [5]. Based on these results, the authors suggested that early HALT after TAVI did not appear to have a significant impact on mid‐term mortality or SVD.

In contrast, we previously encountered a case of HALT accompanied by reduced leaflet motion (RLM) following Perceval (Corcym, London, UK) valve implantation, in which both findings improved after the administration of heparin and warfarin [6]. In the present case, the prosthetic valve exhibiting HALT was also macroscopically confirmed to be a thrombotic valve. These observations suggest that, at least in some cases, HALT could represent an active thrombotic process rather than a purely benign imaging finding. However, given the potential risk of bleeding complications, routine intensification of anticoagulation therapy in all patients with HALT cannot be universally recommended. Both the GALILEO [7] and ATLANTIS [8] trials demonstrated that direct oral anticoagulants reduced HALT after TAVI; neither trial showed an improvement in clinical outcomes. Notably, the GALILEO trial was terminated early due to increased mortality and bleeding in the rivaroxaban group, underscoring that routine anticoagulation solely to prevent HALT cannot be justified.

Sato et al. pathologically evaluated explanted valves after TAVI and classified leaflet thickening into three phases: acute, organizing, and organized [9]. Although differentiation between the acute and organized phases remains difficult based on CT imaging alone, early‐phase HALT might be more likely to respond to anticoagulation therapy. Therefore, when HALT is identified, intensified warfarin therapy (target INR 2.0–3.0) for approximately three months might be useful in selected patients. Furthermore, considering that HALT represents a dynamic and potentially reversible process, in cases showing a rapid increase in transvalvular velocity after SAVR or in those in whom HALT is considered to be in the acute phase, intensification of anticoagulant therapy could be considered after careful assessment of the individual bleeding risk, even if more than six months have elapsed since surgery.

## Author Contributions

**Takanori Kono:** conceptualization, investigation, writing – original draft, writing – review and editing, methodology. **Kazuyoshi Takagi:** conceptualization, writing – original draft, writing – review and editing, methodology, supervision. **Yusuke Shintani:** investigation, methodology. **Hiroki Mine:** investigation, methodology. **Eiki Tayama:** conceptualization, writing – review and editing, supervision.

## Funding

The authors have nothing to report.

## Consent

Written informed consent for publication was obtained from the patient.

## Conflicts of Interest

The authors declare no conflicts of interest.

## Supporting information


**Video S1:** Preoperative three‐dimensional computed tomography reconstruction of the bioprosthetic aortic valve.

**Video S2:** Preoperative transesophageal echocardiographic view of the bioprosthetic aortic valve.

## Data Availability

The deidentified participant data will be shared on a request basis. Please contact the corresponding author directly to request data sharing.
